# Exploring the experience of inpatients with severe alcohol use disorder on a managed alcohol program (MAP) at St. Paul’s Hospital

**DOI:** 10.1186/s12954-020-00371-6

**Published:** 2020-05-12

**Authors:** Beena P. Parappilly, Emma Garrod, Ryan Longoz, Eric Eligh, Holly van Heukelom, Christopher Kit Fairgrieve, Bernadette Pauly

**Affiliations:** 1 Acute Medicine Program, Providence Health Care, St. Paul’s Hospital, 1081 Burrard Street, Vancouver, BC V6Z 1Y6 Canada; 2 Urban Health Program, St. Paul’s Hospital, 1081 Burrard Street, Vancouver, BC V6Z 1Y6 Canada; 3 Addiction Medicine, St. Paul’s Hospital, 1081 Burrard Street, Vancouver, BC V6Z 1Y6 Canada; 4 School of Nursing, University of Victoria, Scientist, Canadian Institute for Substance Use Research, University of Victoria, Box 1700 STN SCS, Victoria, BC Canada

**Keywords:** Alcohol use disorder, Managed alcohol program, Hospital, Patient experience, Harm reduction

## Abstract

**Background:**

Managed alcohol programs are a harm reduction approach for people with severe alcohol use disorder that provide alcohol in a structured setting. We examined the patient experience of receiving alcohol after the implementation of a hospital-based managed alcohol program.

**Methods:**

Using an interpretative descriptive methodology, we conducted interviews with five patients. The criteria for enrollment included continuation of community managed alcohol program or provision of alcohol for stabilization in hospital and ability to provide consent.

**Results:**

Five themes emerged in the analysis: (1) *Reasons for alcohol use* highlighting factors leading to alcohol consumption; (2) *I’m very appreciative* indicating participant’s perception of hospital-based managed alcohol program; (3) *From just vibrating to calm* and *It’s kinda like a pacifier for me* recognizing the impact of hospital-based managed alcohol program on managing withdrawal and on psychological health; (4) *I have no need to go anywhere at all* demonstrating engagement in healthcare; and (5) *Might be nice to have a selection for other people* indicating the need for a broader selection of alcohol.

**Conclusions:**

This study helped to explore the effectiveness of a hospital-based managed alcohol program as experienced by the patients. Overall, participants had a positive experience on hospital-based managed alcohol program. Their perceptions can be used to inform implementation of managed alcohol programs in other hospital settings.

## Background

There are challenges associated with the medical management of people with severe alcohol use disorder admitted to hospital settings, especially individuals experiencing homelessness: patients leaving against medical advice before treatment is completed [1], consuming non-beverage alcohol such as hand sanitizer [2], and leaving the unit and returning to hospital in an intoxicated state [3]. Further, in patients who leave against medical advice, alcohol use has been found to be significantly associated with readmissions within 15 days [4]. Previous research indicates that people who have a history of using non-beverage alcohol such as hand sanitizer experience difficulties accessing medical services, especially when they are intoxicated [5, 6]. Additionally, the treatment and management of patients with severe or illicit alcohol use disorder is complex and challenging for health care providers in the hospital settings [7, 8]. Some patients may benefit from harm reduction approaches that do not require the cessation of alcohol consumption.

The use of beverage alcohol, given in a structured setting called a managed alcohol program (MAP), is a method of harm reduction for people with severe alcohol use disorder (AUD)—especially for those consuming non-beverage alcohol such as hand sanitizer or mouthwash—who have not responded to conventional treatment options. In a number of studies looking at community-based MAPs (CMAP), these programs have been associated with a variety of positive health outcomes including decreased overall alcohol consumption, reduced non-beverage alcohol consumption [9], decreased alcohol withdrawal seizures, and reduced emergency department visits, hospital admissions, and police encounters [10, 11]. It has been calculated that there is a savings of between $1.09 and $1.21 for each dollar invested in MAP due to significant reductions in the frequency of all service utilization by program recipients [12]. MAPs have also been shown to improve the perceived overall quality of life and feelings of safety for participants [13].

There has been limited attention to hospital-based MAPs (HMAP) for those with severe alcohol use problems and homelessness. In a review of the literature on HMAPs that comprised 42 studies on HMAP and MAP, it was identified that MAPs offer health care professionals an opportunity to respond in a respectful and trustworthy manner to the complex needs of people who use alcohol and who experience significant barriers within the healthcare system [14]. Indeed, hospital-based MAP represents a significant opportunity for healthcare providers to engage with patients who may not be benefitting from conventional treatments or who may have had unsuccessful attempts at abstinence [15]. The literature review on HMAPs identified several hospitals that provided alcohol on a short-term basis to manage withdrawal, but none were specifically described as formal managed alcohol programs and there was a noted lack of consistency in assessment and other program components [14]. There is a long history of alcohol being used to manage alcohol withdrawal; US-based studies have determined that 70% of hospital pharmacy formularies surveyed stock alcohol, usually in the form of ethanol [16, 17].

In response to the challenges of managing patients with severe alcohol use disorder and building on the success of CMAP, hospitals have begun implementing this formalized approach. In Canada, there are at least three formal hospital-based managed alcohol programs [18]. Providence Health Care initiated a formal HMAP in July 2016 for acute care inpatients at St. Paul’s Hospital in Vancouver, Canada. The primary goals for introducing HMAP at St. Paul’s Hospital included mitigating the potential harms associated with patients engaging in unsupervised use of alcohol (e.g., excessive or non-beverage alcohol consumption) or leaving hospital against medical advice due to a lack of availability [14, 19], as well as continuing patient’s CMAP dosing if appropriate. This program is available in the emergency department as well, where challenging behaviors have been reported for patients with alcohol use disorder [20]. In this program, alcohol is prescribed by the Addiction Medicine Consult Team for patients who are on a CMAP or for patients who regularly consume alcohol and/or non-beverage alcohol and are at risk of experiencing withdrawal and unlikely to benefit from traditional medical management of withdrawal. Alcohol is prescribed using a customizable order set, which includes scheduled blood work, vital signs, and monitoring of alcohol intoxication or withdrawal symptoms. Additionally, these orders specify the dose, frequency, and type of alcohol (vodka or beer) to be administered. Orders are written so that alcohol is to be administered as needed, with specified minimum time limits between doses and not to exceed administration of 18 doses/24 h. Doses are given at patient request or offered if the patient is showing signs of withdrawal. Nurses who are administering alcohol must assess the patient for obvious signs of intoxication before dispensing a dose. If patients appear intoxicated, the dose must be held and the Addiction Medicine Consult Team notified.

While there is a growing body of literature in support of CMAPs, there are very few published articles regarding HMAPs and, to our knowledge, no published study regarding the patient experience of receiving managed alcohol in the acute care setting. One of the recommendations from a review of HMAPs was to determine the safety of HMAPs and effectiveness in terms of ensuring patients stay in hospital to complete medical treatment [14]. Qualitative inquiries can provide understanding of why particular impacts occur from the patient’s perspective [21], which is essential in novel programming and a nascent literature base. Qualitative explorations of CMAPs have yielded important insights into reasons for program successes that include positive psychological outcomes that promote health and recovery [22] and have been recommended as a way to explore the patient experience with HMAP [23]. In this study, we aimed to capture the insights of patients with AUD while on HMAP, to help tailor our delivery of the program and to relay insights that may guide other hospitals who are considering implementing HMAP to ensure that this promising intervention is delivered in a safe, effective, and patient-centered manner. Specifically, we sought to answer the question, “What is the experience of patients with severe alcohol use disorder on a managed alcohol program (MAP) while admitted to an acute care hospital?”

## Methods

### Design

A qualitative methodology was employed for this study in order to gain an understanding of patient experiences with a HMAP. Interpretive description [21] was utilized to guide the multi-step data analysis that took place to facilitate a qualitative inquiry into the care experience of inpatients receiving MAP. Interpretive description considers the value of subjective and experiential knowledge and the time and context that provide clinical insight into understanding of human experiences [21]. Interpretive description also acknowledges that human experiences are socially constructed and that there are multiple constructed realities to human experience. The purpose of interpretive description is not to generate theory but to provide insights into practice-based issues and make recommendations for practice. Given that HMAP is a novel initiative, interpretive description is ideal for exploring the care experience of participants and making recommendations for practice improvement.

### Participants

Participants were recruited from in-patient units at St. Paul’s Hospital; each study participant had been assessed by the Addiction Medicine Consult Team and initiated on HMAP on an individualized basis. While strict inclusion and exclusion criteria for MAP initiation at St. Paul’s Hospital have yet to be finalized, indications for HMAP include continuation of CMAP or to stabilize outside alcohol use in order to improve retention in hospital and completion of medical treatment. In addition, study participants were all 19 years of age and older, able to communicate in English, competent to provide informed consent, and medically stable to participate in the interview. Recruitment was initially intended to take place over a 6-month timeframe. Due to low enrolment numbers as a result of the infrequent initiation of HMAP, however, a 6-month extension was granted, making the recruitment period a total of 1 year in length.

### Data collection

A semi-structured qualitative interview tool with open-ended questions was adapted from a previously published tool used with participants of a CMAP [24]. The modified interview tool was pilot-tested with a patient on HMAP and adjusted to improve the wording and flow of the interview. Prior to all interviews, the Capacity Assessment Instrument for People Who Misuse Substances, created by the University of British Columbia [25], was adopted to determine participants’ ability to provide consent. When interested patients were discharged from hospital prior to being interviewed, consent was obtained for a research assistant to contact the patient outside of the hospital to set up a meeting as soon as possible. All participants were provided with a consent letter outlining the details of the study, including the research assistant’s contact information should the patient have required further information or clarification. This consent form was reviewed with the patient, and any questions the patient had were answered by the research assistant before signing. The potential participants were told that participation in this study would not impact their care. Interviews were between 17 and 47 min in duration, and each was conducted by a research assistant in a private setting. Four of the five participants were interviewed while still admitted to hospital, whereas one participant was interviewed at a nearby supportive housing facility 2 days after discharge. Participants were provided a $20.00 cash honorarium for their involvement in the study. All interviews were recorded and transcribed verbatim by a hired transcriptionist. Ethics approval was obtained from the Providence Health Care Research Ethics Board associated with the University of British Columbia. Pseudo names have been used to identify the participants in this study.

### Data analysis

First, all transcribed interviews were entered into a Microsoft Excel spreadsheet and reviewed line-by-line for meaning, events, interactions, and actions as described by the participant. The first four interviews were then divided in two, and members of the research team, working in two separate pairs, assigned emerging themes and sub-themes to each half of the data. Emerging themes and sub-themes were then reviewed together by the opposite pairs of the research team in order to provide consistency and improve inter-rater reliability. This process helped us to develop a code book with the codes that were agreed upon by the research team. This code book was used to code the fifth and final interview. As this process did not produce any new codes, the final stage of coding consisted of grouping thematically relevant concepts leading to the results. Immersion in the data of individual cases helped the researchers thoroughly understand each individual case and generate common themes. The research team, including a qualitative research expert, reviewed the codes, subthemes, and themes in the last phase to ensure alignment and that the themes reflected the overall experience of the patients on HMAP.

### Trustworthiness

To confirm trustworthiness in this study, two strategies were utilized: triangulation and reflexivity. A qualitative research expert provided training on qualitative research at the beginning and throughout the project and also provided guidance on data analysis and the codebook. The research team also had regular discussions about the data collection experience and analysis which ensured reflexivity in the study. Lastly, field notes and memos were maintained during the research process to (a) reflect on the experience, (b) ensure sufficient information would be available to assist others to understand the analytic reasoning process, and (c) demonstrate that the analysis was grounded within the data. An audit trail was kept to document decision-making related to finalizing the coding tree.

## Results

During the data collection period of April 2018 to March 2019, there were 11 potential participants for this study. Of the 11, there were five in-patients receiving MAP who consented to participate in the study. The remaining six inpatients were not interviewed for the following reasons: three left against medical advice either before being consented and/or interviewed, one was discharged before being approached, one declined to participate, and one did not meet the inclusion criterion of cognitive capacity. Refer to Table 1 for a description of the five participants.

**Table 1:** Demographic characteristics of participants, *n* = 5

Age in years (mean, range)	45, 25–58
**Identified gender (*n*)**	Male 2, female 3
**Identified ethnicity**	Indigenous 4, Caucasian 1
**Marital status**	Single 3, partner 1, widowed 1
**Housing status**	Supportive housing 2, no fixed address—shelter 2, no fixed address—other 1
**Education**	Grade 7, 1; grade 9, 2; college/university, 2
**Employment**	Persons with disability (PWD) 3, income assistance (IA) 1, Canada pension plan (CPP) 1
**Hospital length of stay in days (mean, range)**	16, 7–28

Of the five participants, only one participant reported receiving HMAP during a prior hospitalization. Further, only one participant received CMAP prior to admission, whereas another participant received regular dosing of alcohol as prescribed by a physician at their supportive housing residence. During the hospitalization of these five participants, the average number of days on MAP was 14 with a range of 6 to 22 days. Four of the five participants were started on MAP within 1 day of admission. The HMAP included vodka or beer; however, one participant was prescribed wine as there was a small stock remaining from when wine was part of the order set. Three of the five participants were documented to have consumed additional alcohol outside the program; however, none of the participants were documented to have consumed non-beverage alcohol during this time. One participant left the hospital against medical advice after an 11-day hospitalization, and another participant’s MAP was discontinued a few days before their discharge, after their medical treatment and a discharge planning discussion were both completed. HMAP was prescribed and administered as described in Table 2.

**Table 2:** Hospital MAP prescription and administration (*n* = 5)

Participant	MAP1	MAP2	MAP3	MAP4	MAP5
**MAP prescription**	Beer (355 mL) orally every 1 h as needed to maximum of 9 doses/24 h	Beer (355 mL) orally every 1 h as needed to max of 10–12 doses/24 h*	Red wine (200 mL) orally every 3 h as needed to max of 4 doses/24 h	Vodka (50 mL) orally every 1 h as needed to max of 8 doses/24 h	Vodka (50 mL) orally every 1 h as needed to max of 12–18 doses/24 h*
**MAP doses administered (mean/24 h)**	8.6	9.8	2.9	7.0	8.5

*Dose increased during stay to retain patient in hospital and reduce consumption of non-MAP alcohol

Analysis revealed five main themes which are discussed below: reasons for alcohol use, patients’ perception of HMAP, impact of HMAP on managing withdrawal and on psychological health, engagement in health care, and opportunities for improvement.

### Theme 1: reasons for alcohol use—“I lost it”

Participants cited several different factors associated with impulses to drink: financial or reward-based, physical, social, and emotional, related to themes of loss and hurt. The most significant factors leading to impulses to drink, especially to intoxication, were emotional. Diane, for example, shared her reasons for drinking alcohol: Diane: “lost my older sister, my second youngest brother, and… helped bury my baby sister... I lost it and I just went nuts and started drinking, that hand sanitizer….” She also described racist encounters with neighbors that left her angry and hurt, as well as frustrating encounters with the healthcare system not meeting her needs. Similarly, Stephen also recalled binges triggered by loss: Stephen: “I lost a couple of friends and, so I get into a pity pot. And, I drank straight rubbing alcohol for almost five days.” Physical triggers such as cravings or tremors were also noted to lead to impulses to drink. Stephen: “I go through the tremors really bad in the morning, and so, if I can have a couple of shots, it really helps.” Participants demonstrated significant insight into precursors, their underlying causes, and mitigating factors, citing elements such as stable housing as factors in drinking less. The responses of these participants would challenge many healthcare providers’ assumptions about people with severe alcohol use disorder and prompt us to understand their behavior within a broader context of harms and seek a strengths-based understanding of patient’s lived realities.

### Theme 2: patients’ perception of HMAP—“I’m very appreciative”

Four out of five participants in this study self-identified as indigenous and largely had positive descriptions of the care received. Diane: “It’s to help me not go through some of the acute withdrawal…and, it stabilized me right now. I’m comfortable; I don’t feel antsy or anxious… “I need my beer!” It’s just - I’m cool with the way they’re, they’re serving me this stuff. I’m very appreciative.” The novelty of the provision of alcohol in a hospital setting was appreciated by all participants. Diane: “I was shocked, I was like ‘Oh my God…I’m drinking beer in a hospital!” Another participant, Jennifer also commented on this novelty: Jennifer: “Oh, I’ve enjoyed it. I’ve been a butterfly! Just coasting in the air, feeling light and loved and important to feel that. And I’ve never had that experience before, so it’s a new thing, you know. I’m surprised the hospital actually getting involved in that, but I’m pretty sure it will go a long ways.” Their expressed “shock” at the program indicates how it differed significantly from past experiences.

While transitioning from CMAP to HMAP, participants noted the significant difference between these two programs. Diane, for example, shared her observation about the two programs: Diane: “It’s basically… [a] more calmer, safer environment for me. And the team work here really helped me comfort and they’re really good at explaining the situations that I’m pacing and stuff like that.” Diane also appreciated the opportunity for increased socialization and feeling less stressed in the hospital environment stating: Diane: “Whereas at home I’d be stressed out. But I still get my beer. Might be in my room alone – I have company here, at least …and the comfort of the teamwork with you and the other people.”

While discussing the differences between HMAP and CMAP, some participants focused on the dosing schedule and its flexibility: Stephen: “It’s been good. I know I’m allowed eight bottles a day…I’m allowed one every hour if I want. And, it also helps me spread it out a bit. When I feel my body starting to go into a bit of the tremors, and stuff like that, then I’ll go and ask the nurse for another bottle. I try not to take it every hour and, so far, I haven’t even filled my quota. So, I think yesterday was seven bottles, I think the day before was something like six. So, and today I’ve had two and, so, it just, it helps calm everything down.” Stephen also commented on the availability of alcohol choices: Stephen: “Well, we don’t do hard liquor on the other one. So, there’s no hard liquor; it’s just wine and beer that we get to replace our hard liquor with.” This insight raises the important issue of alcohol selection as a factor in HMAP’s accessibility, which will be discussed in greater depth in subsequent sections.

### Theme 3: impact on health

#### 3.1 Managing withdrawal—from just vibrating to calm

Most participants commented on how HMAP was effective in relieving their withdrawal symptoms and promoted a more relaxed experience for them. This is conveyed in the words of Diane: “I can’t put it into detailed words but…I’m really happy you guys have this here…when I came here, I was just vibrating, but in two days I was calm. I’m still in lots of pain but I look forward to this cause it was like, you know, a thin blanket or a band aid sort of thing. No, not band aid, just something, a coating to help soothe everything that’s going on in me... there’s not enough words I can describe to, for how much this program is helping me here.”

Some participants found HMAP superior to taking medications such as lorazepam (Ativan) or diazepam (Valium) which are commonly used to treat alcohol withdrawal symptoms: Stephen: “I go through the tremors really bad in the morning, and so if I can have a couple of shots, it really helps with my tremors – better than, say with Ativan or Valium or any of those that don’t really do much for my tremors. They make me dopey, but, I find a couple of shots settle me down.” Similarly, both Robert and Jennifer recalled how HMAP helped them:

Robert: “well, it helps you feel better. And it doesn’t make you feel sick”

Jennifer: “pills aren’t always the answer”

These statements highlight the fact that the participants in this study experienced benefits in the management of their withdrawal symptoms. Although MAP is not explicitly used for withdrawal, it can be used in this regard for individuals who do not benefit from conventional treatment.

#### 3.2 Impact on psychological health—“It’s kinda like a pacifier for me”

While discussing the experience of HMAP, most participants talked about how the treatment provided them with some measure of psychological comfort:

Diane: “…they reassured me and they gave me this, so it’s kinda like a pacifier for me”

Sarah: “it calms me down when I’m being scared.”

HMAP was also reported to ease anger and aggression as stated by Sarah: “When I was here and I wasn’t drinking for 2 days, I was… getting really irritated and I was getting really aggressive and mean. So, now they have me on the vodka and I’m very calm.” Participants commented on their feelings of security and satisfaction with HMAP, for example, Diane stated: “every day I get nine and I don’t feel antsy or, “I need a beer!” It’s just I know when I wake up it’s there and I’m really happy with that.” Similarly, Sarah talked about the relief in knowing that alcohol would be available: Sarah: “Every time I take another shot…I still look out the window, still in pain but I know that I have alcohol waiting for me so – I can still look out the window and… I feel comfortable. But I am still in pain. But alcohol does help a lot and especially just that little shot helps.” The sense of emotional relief expressed by the participants suggests that HMAP can be quite effective in mitigating the numerous psychological stressors within the hospital environment and related to experiences of illness.

### Theme 4: engagement with the treatment plan—“I have no need to go anywhere at all”

Most participants indicated that receiving MAP in the hospital met their needs, and as a result, they found no reason to leave the hospital before their medical treatment was finished. HMAP was identified as an incentive for them to stay in the hospital: Diane: “I have what I need here so I have no need to go anywhere at all. Till I get proper discharge.” Diane also expressed how HMAP helped to reduce her fear of being at the hospital and continuing to stay and receive care: Diane: “I don’t want to stay in. I don’t like hospitals personally but, because of my health issues and how they’re helping me with the MAP… I’ll do whatever’s needed to be. Cause I just – I don’t want to die… All I know is I don’t want to die.”

Sarah, who previously had a negative experience with not having her withdrawal needs met, stated how HMAP helped her to stay engaged in care: Sarah: “It’s helping me stay here. It’s helping me find out what the fuck’s wrong with me. I’m kinda scared. And it’s been helping me to stay and go through all the tests and stuff.” Sarah also described how HMAP helped her to feel settled and not get angry, explaining, “I don’t get angry, I don’t want to leave. I feel settled.” Similarly, Jennifer recalled how MAP helped her: Jennifer: “How has MAP helped me stay in hospital?...Help to stay in the hospital, yes, that’s the whole point. It just basically I agree with their terms and it’s joy.” Having access to HMAP was also reported to help manage cravings and the impulse to leave: Stephen: “I really thought about going for a walk down at the liquor store the other day and getting myself my own personal mickey. And, I talked myself out of it. I thought, no, let’s do this. So, there’s things like that, I mean there’s liquor stores everywhere, you don’t have to go far, even if you’re in a blue robe.” These findings are promising from the patient perspective, but should also be seen as an opportunity for health care providers who may have historically struggled with the complexities of engaging and managing the needs of patients who use alcohol.

### Theme 5: opportunities for improvement—“Might be nice to have a selection for other people”

The managed alcohol program in our hospital included beer and vodka. The selection of the type of alcohol depended on what patients were using in the community and in consultation with the patient. One patient was served wine as there was a small stock of wine remaining after its discontinuation as an option for the HMAP. Since this program was new to our organization, participants were asked for suggestions to improve the program and they offered several insights. Their suggestions were related to dosing, choices of alcohol, and frequency:

Sarah: “they’ve been giving me vodka – I really don’t like it.”

Stephen: “I’m a vodka drinker and that’s what they give me so, it’s kinda my thing anyhow, right? If somebody is drinking, you know, whiskey or rum or something like that, might be nice to have a selection for other people” and suggested the program could be improved “if we had a choice of what [type of alcohol] we wanted.”

Nursing workload and scheduling issues were cited as reasons for waiting for doses. Sarah stated, “Shift change around 7 o’clock, it takes a while. Like I don’t mind, I don’t mind waiting but it just kind of sucks. It’s just I’m really impatient.” Another participant, Robert expressed that the administration of alcohol would be better if nurses “[gave] it to me regularly,” whereas doses are ordered to be given only on an as-needed basis. These suggestions inform our analysis and subsequent recommendations for improving future HMAP patient experiences.

## Discussion

In this study, we explored and reported on the insights and experiences of patients with AUD receiving managed alcohol as a harm reduction strategy in hospital. Provision of a MAP in an acute care setting is a novel approach used to treat patients with severe AUD who decline or have not responded to conventional treatment and whose alcohol use is interfering with medical care. The findings from this study show that participants are very appreciative of the program and show significant insight into reasons for consuming alcohol and the benefits of HMAP, physically, psychologically, and endorsing its role in enhancing their engagement in treatment. These findings offer meaningful feedback about the program and highlight opportunities to improve care for these highly marginalized patients.

Participants report that HMAP was effective in supporting psychological safety aligns with research findings that CMAP improves the overall quality of life and feelings of safety for program participants [13]. The review by Brookes et al. [14] recommended evaluating HMAPs safety and impact on patient’s completion of treatment; it is important to note that one of the salient findings of our study is that HMAP helps patients stay engaged with their respective treatment plans and remain in hospital. Further, the availability of an HMAP can serve as a bridge to care for patients receiving CMAP who require admission to and retention in hospital. The study findings indicated that HMAP participants felt their needs were being met, which deterred them from going elsewhere to use alcohol and to instead remain in hospital and engaged in care. These findings are similar to previous literature [22], which stated that MAP played an important role in dealing with the urges to drink among participants in a community setting giving them a sense of control over their drinking. The participants generally appreciated alcohol being available to them and the flexibility of dosing schedules during their stay, which may also have helped them to focus on medical treatment and stay in the hospital.

Some participants noted a preference for managing their withdrawal with alcohol as opposed to benzodiazepines. Although this is not the goal of HMAP or the traditional approach to managing acute alcohol withdrawal, this preference may be part of what engaged patients in care. Of note, no participants reported use of non-beverage alcohol during their time on HMAP, whereas two participants reported they had consumed non-beverage alcohol in the past prior to hospitalization. Given the deleterious effects of non-beverage alcohol on health and function, this can be considered a positive shift in consumption patterns.

The positive experiences reported by participants in this study run counter to accounts of stigmatizing and harmful encounters with the healthcare system described by people who use alcohol, specifically non-beverage alcohol [5], and in particular by individuals who identify as indigenous [6], as well as accounts by this study’s participants of past negative occurrences in hospital settings. Four out of five participants in this study self-identified as indigenous and largely had positive descriptions of the care received. Although the impact of this program on the experiences of indigenous participants was not explicitly explored in this study, it is important to note that in Canada, indigenous people are over represented among those with severe alcohol use disorder [13]. Several CMAPs across Canada incorporate indigenous wellness activities [26], and St. Paul’s Hospital has an Indigenous Health Team that provides cultural support to patients. Hospital staff have also found benefit in this program; nurses surveyed in the same setting (*n* = 97) generally supported harm reduction and thought the benefits of HMAP outweighed the risks [27]. Indeed, hospital-based MAPs offer a meaningful opportunity for the healthcare system to meet the needs of individuals who have largely been excluded from healthcare settings [6] and improve health outcomes.

Given the lack of extant literature on HMAP and the opportunity to solicit feedback on a program, the study team wanted to know if there were opportunities for improvement. Participants brought up alcohol selection and dose timing. At St. Paul’s Hospital, the MAP protocol provides doses on an as-needed basis, meaning a patient needs to ask for a dose or a nurse may offer if the patient is exhibiting withdrawal symptoms. Patients suggested having some regularly scheduled doses might keep them more comfortable. In terms of selection, at the time of the study, beer and vodka were the two options on the order set with wine having been just discontinued due to minimal ordering. An inventory audit conducted in June 2019 revealed that beer and vodka were administered equally. Recently in British Columbia, both vodka and beer were approved for provincial drug formulary, meaning it can be available through hospital pharmacies, demonstrating support for this evidence-based harm reduction approach. As the participants in this study noted, selection matters to their perception of the care being offered and their sense of well-being.

### Strengths

The main strength of this study is that it explored the effectiveness of HMAP as experienced by the patients and is the first published study to our knowledge exploring the patient experience on HMAP. It provided a deep reflection of the patient experience through their descriptions of how they felt about being on HMAP. The findings were derived from the data provided by the patients and reflect the current practice of how HMAP is delivered. Another strength of this study is that the findings were immediately used to lobby for introducing alcohol choices for HMAP into the drug formulary at the provincial level, demonstrating rapid knowledge translation and application. Lastly, the study team was multidisciplinary by design and included point of care nurses, a nurse educator, a clinical nurse specialist, an addiction physician, and a patient care manager, with mentorship from an established research scientist, to elicit a comprehensive understanding of the patient experience of HMAP and broadened possibilities for utilization of findings.

### Limitations

The transferability of this study’s findings is limited due to its small sample size of a unique patient population that may find it difficult to remain engaged in hospital care due to a variety of unfortunate factors such as stigma, previous experiences with under-treatment of withdrawal, or perceived substandard care. Another limitation is that this study was conducted in one tertiary hospital, and thus, findings are limited to hospitals of similar nature. In addition, the team had also wanted to explore the experience of patients as they transitioned from CMAP to HMAP, but most patients were not on a formal CMAP prior to admission. Lastly, the participants were interviewed by study team members who are hospital staff and may have felt they could not say anything negative despite the reassurance from the study team that participation in the study would not impact their care.

## Conclusions

This study explored the experiences of patients who received managed alcohol as a harm reduction approach for alcohol use disorder while admitted to hospital. To summarize, this study demonstrates that patients overall had a positive experience on a hospital-based managed alcohol program and described its benefits in managing withdrawal, engagement in care, and overall feelings of psychological and physical safety while receiving care in hospital. More research is needed to obtain a comprehensive understanding of the benefits of hospital managed alcohol programs on the physical, mental, and social wellbeing of patients with alcohol use disorder in hospital. In addition, further study is required to determine the optimal type and dosing of alcohol for effective management and engagement of patients with severe alcohol use disorder in hospital settings. The voices of these patients, heard through the study findings, can be used to inform implementation of future managed alcohol programs in other hospital settings.

## Data Availability

The datasets used and /or analyzed during the current study are available from the corresponding author on reasonable request.

## Abbreviations

MAP: Managed alcohol program
AUD: Alcohol use disorder
CMAP: Community-based MAP
HMAP: Hospital-based MAP
